# Anterior cruciate ligament repair – past, present and future

**DOI:** 10.1186/s40634-018-0136-6

**Published:** 2018-06-15

**Authors:** Piyush Mahapatra, Saman Horriat, Bobby S. Anand

**Affiliations:** Trauma and Orthopaedic Department, Croydon University Hospital, 530 London Road, London, CR7 7YE UK

**Keywords:** Anterior, Cruciate, Ligament, Primary, Repair, Reconstruction, Athletes

## Abstract

**Background:**

This article provides a detailed narrative review on the history and current concepts surrounding ligamentous repair techniques in athletic patients. In particular, we will focus on the anterior cruciate ligament (ACL) as a case study in ligament injury and ligamentous repair techniques. PubMed (MEDLINE), EMBASE and Cochrane Library databases for papers relating to primary anterior cruciate ligament reconstruction were searched by all participating authors. All relevant historical papers were included for analysis. Additional searches of the same databases were made for papers relating to biological enhancement of ligament healing.

**Current standard:**

The poor capacity of the ACL to heal is one of the main reasons why the current gold standard surgical treatment for an ACL injury in an athletic patient is ACL reconstruction with autograft from either the hamstrings or patella tendon. It is hypothesised that by preserving and repairing native tissues and negating the need for autograft that primary ACL repair may represent a key step change in the treatment of ACL injuries.

**History of primary ACL repair:**

The history of primary ACL repair will be discussed and the circumstances that led to the near-abandonment of primary ACL repair techniques will be reviewed.

**New primary repair techniques:**

There has been a recent resurgence in interest with regards to primary ACL repair. Improvements in imaging now allow for identification of tear location, with femoral-sided injuries, being more suitable for repair. We will discuss in details strategies for improving the mechanical and biological environment in order to allow primary healing to occur.

In particular, we will explain mechanical supplementation such as Internal Brace Ligament Augmentation and Dynamic Intraligamentary Stabilisation techniques. These are novel techniques that aim to protect the primary repair by providing a stabilising construct that connects the femur and the tibia, thus bridging the repair.

**Bio enhanced repair:**

In addition, biological supplementation is being investigated as an adjunct and we will review the current literature with regards to bio-enhancement in the form platelet rich plasma, bio-scaffolds and stem cells. On the basis of current evidence, there appears to be a role for bio-enhancement, however, this is not yet translated into clinical practice.

**Conclusions:**

Several promising avenues of further research now exist in the form of mechanical and biological augmentation techniques. Further work is clearly needed but there is renewed interest and focus for primary ACL repair that may yet prove the new frontier in ligament repair.

## Review

Ligamentous injury in the athlete is a major cause of morbidity and time away from sport (Waldén et al. 2001, 2016; Brophy et al. 2012; Lundblad et al. 2013). Ligamentous repair remains an ongoing aspiration in the treatment of athletic patients in order to try and facilitate a rapid and complete return to high level sporting activity.

The vast majority of ligamentous sporting injuries in athletes affects either the ankle or the knee joint (Darrow et al. 2009; Kerr et al. 2011; Rechel et al. 2011; Swenson et al. 2013). Although, the ankle is more frequently injured than the knee, knee injuries are the leading cause of sport-related surgery (Joseph et al. 2013) and knee ligament injuries can have devastating consequences on the sporting career of athletes. In particular, we will focus on the anterior cruciate ligament (ACL) as a case study in ligament injury and ligamentous repair techniques. PubMed (MEDLINE), EMBASE and Cochrane Library databases for papers relating to primary anterior cruciate ligament reconstruction were searched by each author. All relevant historical papers were included for analysis. Additional searches of the same databases were made for papers relating to biological enhancement of ligament healing.

We will describe ‘primary repair’ as any surgical procedure that involves restoring the original native injured ligament. If the procedure involves introducing a graft to replace the original injured ligament we will refer to this as a reconstruction. It is important for the reader to be clear of the differences as ‘repair’ is often used incorrectly within the literature to describe reconstruction techniques.

## Anterior cruciate ligament injuries

Anterior cruciate ligament injuries account for anywhere between 25 and 50% of ligamentous knee injuries (Risberg et al. 2004) and pose unique clinic problems because of its poor capacity to undergo biological healing due to the local intra-articular conditions. A potential theory to explain this is that the synovial fluid and intra-articular movement prevents formation of a stable fibrin-platelet scaffold (Murray 2010). Without this scaffold, no primary healing can take place (Murray et al. 2000).

This poor capacity of the ACL to heal is one of the main reasons why the current gold standard surgical treatment for an ACL injury in an athletic patient is ACL reconstruction with autograft from either the hamstrings or patella tendon. The results of ACL reconstruction are good (Lai et al. 2017) but current techniques do pose their own challenges and potential issues. These include decreased hamstrings strength (Holsgaard-Larsen et al. 2014; Konrath et al. 2016; Setuain et al. 2016), anterior knee pain (Xie et al. 2015) and loss of proprioception (Zhou et al. 2008) There is also significant evidence to suggest that ACL reconstruction does not prevent future osteoarthritis (Ajuied et al. 2014; Adravanti et al. 2017).

So, is there a better solution? It stands to reason that by preserving and repairing native tissues and negating the need for autograft that primary ACL repair may represent a key step change in the treatment of ACL injuries. In particular, negating the requirement for autografts would theoretically solve troublesome donor site morbidity issues such as loss of hamstrings strength and anterior knee pain. Current practices and trends towards remnant preservation and some of the improvements shown in subjective proprioceptive outcomes, knee stability and revision rate (Takazawa et al. 2013; Takahashi et al. 2016; Muneta and Koga 2017; Andonovski et al. 2017) can be extrapolated to offer hypothetical benefits for primary repair over reconstruction.

The interesting question that now arises is that if ligament repair has theoretical advantages over reconstruction then why is it that reconstruction is the current gold standard? In order to be able to answer this question it worth considering the history of primary ACL repair and how we have got to the present-day situation.

### History of primary ACL repair

ACL injuries were apparently first described by the Ancient Greeks (Davarinos et al. 2014). The first primary ACL repair was reported in 1895 by Mayo Robson (van der List and DiFelice 2016). He describes reattaching both cruciate ligaments from their femoral attachment sites using catgut ligatures. Primary ACL repair was refined further and eventually open primary ACL repair became the gold standard for ACL treatment in the 1970s and 1980s (England 1976; Feagin and Curl 1976; Weaver et al. 1985; Sherman and Bonamo 1988).

Although initial results for primary open ACL repair were positive (England 1976; Weaver et al. 1985; Sherman and Bonamo 1988) significant issues began to materialize at mid-term follow up with re-rupture rates of > 50% being reported at 5 years (Feagin and Curl 1976). In addition, ACL reconstruction was being developed and several randomized controlled trails were showing improved outcomes with reconstruction versus primary repair (Andersson et al. 1991; Engebretsen et al. 1990; Grontvedt et al. 1996). As a result, by the 1990s open ACL repair was almost completely abandoned in favour of ACL reconstruction.

However, it is worth understanding that this paradigm shift was complicated by other factors, which are well highlighted by van der List and Di Felice (2016). They state that there were a variety of factors that came together to cause the shift from primary repair to reconstruction. In particular, the key issues to note include primary repair originally being developed and refined as an open procedure with resultant morbidity from the arthrotomy itself. Arthroscopic techniques only became more advanced and refined in the 1990s; once primary repair had already been abandoned.

Additionally, rehabilitation protocols have changed significantly with early mobilization again being developed after the abandonment of primary repair.

Van der List and Di Felice are also critical of Feagin and Curl’s work, which is often quoted as evidence against primary repair. In particular, the now obsolete surgical technique including the use of figure-of-eight absorbable sutures secured over the iliotibial band was thought to be a contributing factor to the poor outcomes reported by Feagin and Curl 1976.

Finally, and perhaps most importantly, much of the early work regarding ACL repair did not take into account tear location. We now know that tear location has a significant bearing on the outcome of primary ACL repair (Sherman et al. 1991). Many of the large randomised controlled trials comparing repair with reconstruction do not take into account this factor (Drogset et al. 2006). Sherman et al. (1991) showed that “poor tissue quality, typical of mid-substance tears” had much poorer results than type 1 (proximal tears) which trended towards better results with primary repair. However, much of the work done before 1991 do not stratify their results with relation to tear location and thus a significant degree of confounding is introduced into these studies (Strand et al. 2005; Meunier et al. 2006).

All of these factors appear to have contributed to the near total abandonment of primary ACL repair with no new cohorts of patients being studied for nearly two decades. It is only within the last two years that there has been a significant increase in interest for primary ACL repair with new case series being published (van der van der List and DiFelice 2016; Achtnich et al. 2016).

### New primary ACL repair techniques

Although reconstruction is currently the gold standard, primary repair, if successful, can theoretically lead to a significant improvement in the treatment of ACL injuries in the athlete. In particular, the improvements in retention of proprioception and native kinematics could be a significant advancement.

Novel techniques for primary ACL repair have developed considerably in recent years (Kohl et al. 2014; MacKay et al. 2015) and now employ the full gambit of advanced arthroscopic techniques currently available. In addition, the improvements in Magnetic Resonance Imaging (MRI) has meant that we are now able to accurately delineate tear location and thus identify those patients who are most likely to benefit from primary ACL repair (Daniels et al. 2018; van der List et al. 2018).

A recent case series (DiFelice et al. 2015), although small (*n* = 11), does show good results at medium term follow up with only one reported re-rupture following primary repair of proximal ACL tears. A further case control study (Achtnich et al. 2016) compared 20 patients with proximal ACL tears that had primary arthroscopic repair with 20 patients with proximal ACL tears that had single bundle ACL reconstruction. They reported excellent stability testing and patient reported outcome in both groups but there was a significantly higher revision rate (15% vs 0%) in the primary repair group.

Therefore, it appears that primary ACL repair is a potential treatment option in specific patients with proximal ACL tears. However, a revision rate of 15% is still not satisfactory and not entirely dissimilar to results from many years ago that led to the near-abandonment of primary ACL repair. So can anything be done to improve these results or is history doomed to repeat itself?

Principles of osteosynthesis dictate that bone healing in fractures needs a suitable mechanical and biological environment to occur and that our aim as orthopaedic and trauma surgeons is to try and provide that environment through whatever techniques and implants are required. It surmises that a similar principle should apply for soft tissue injuries, such as ACL tears. The optimum environment is yet to be identified, but it is evident, as with fracture healing that an element of mechanical stability (Murray et al. 2010a; Seitz et al. 2013) and favourable biology (Mastrangelo et al. 2010; Murray et al. 2010b) are pre-requisites.

As discussed previously, primary ACL repair, as a surgical procedure, has not gone through significant development and refinement and there remain several unanswered issues. A variety of additional techniques and adjuncts have been used in order to try and improve the outcomes and reduce re-ruptures compared to the techniques originally first described in the 1970s and 1980s. Many of these focus on being able to create a satisfactory mechanical and biological environment to allow healing to occur.

## Mechanical stability

Animal studies (Fleming et al. 2008) showed that repairing a torn ACL to the tibial stump, does not improve sagittal plane laxity intra-operatively. The likely reasons for this are due to the inherent difficulties in placing a stitch in a short ligament stump composed primarily of longitudinal fibres. Even with grasping suture techniques there is likely to be significant suture sliding along longitudinal fascicles. However, anchoring of the suture to the tibial ACL footprint, particularly centrally/anteriorly did restore sagittal plane laxity, thus suggesting that a suture bridge from the tibial to the femoral side is crucial in restoring and maintaining early sagittal plane stability.

Additionally, porcine models have demonstrated increased strength with non-absorbable sutures (Vavken et al. 2013). Subsequently, it was found that augmenting the primary ACL repair with a polyethelene tape in a sheep model yielded improved biomechanical properties of the repaired ACL in the form of increased tensile strength and graft stiffness (Seitz et al. 2013). It is perhaps, these studies and ideas that have led to the development of two new techniques in ACL repair. Both involve the use of a non-absorbable polyethylene tape / wire to bridge the repaired ligament from the femoral to the tibial side.

### Internal brace ligament augmentation (IBLA)

**Fig. 1:**
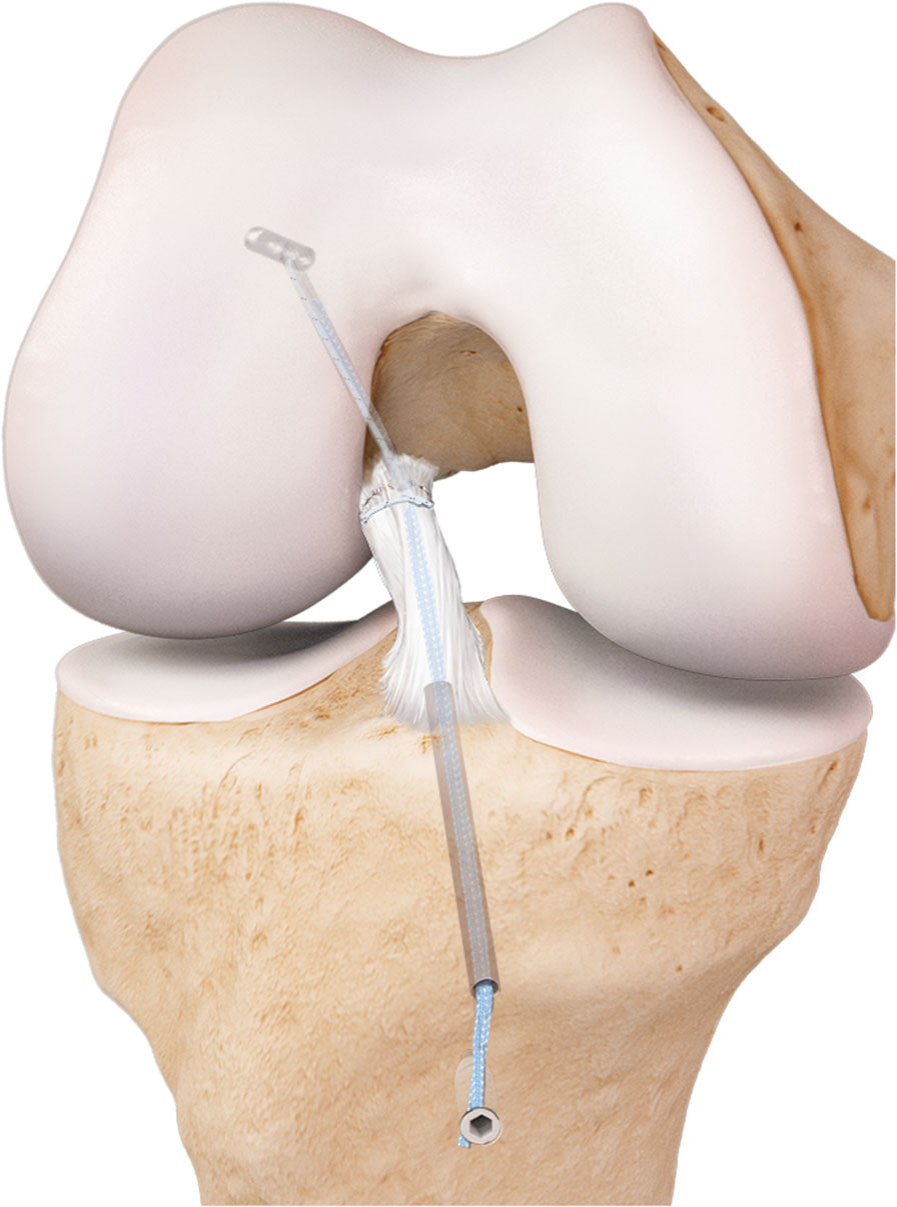
Internal Brace Ligament Augmentation (Arthrex™)

Internal Brace Ligament Augmentation (IBLA) involves using a 2.5 mm polythethylene tape to bridge from the anatomical attachments of the mid-bundle positions of the ACL on both the femur and the tibia (Fig. 1). Extensive micro-fracturing is then carried out on the femoral side to help stimulate biological healing.

Mackay et al. (2015) have performed 68 acute ACL repairs with IBLA since 2011. They describe acutely repairing (within 3 months of injury) femoral-sided avulsions (mid substance tears were reconstructed) with the IBLA technique. A whipstitch was passed through the ligament. The whipstitch and internal brace were secured on the proximal side with the ACL tightrope (Arthrex, Naples, Florida) and distal fixation was achieved with the SwiveLock Suture Anchor (Arthrex, Naples, Florida). Tensioning of the internal brace was carried out with the knee in extension.

They demonstrated similar patient related outcome scores (PROMS) to traditional ACL reconstruction techniques at one year follow up. The re-intervention rate was 6% but there was only 1 failure (re-intervention rate for failure 1.5%). The failure occurred at 18 weeks after return to full contact sports. No additional imaging was carried out to ascertain whether the repair was successful but in the other cases that required re-intervention (stiffness, recurrent meniscal pathology and patellofemoral osteochondral lesion) the repairs were all found to be intact.

Therefore, although a limited series, it appears that IBLA is an interesting potential solution to the high failure rates previously associated with ACL repairs. It stands to reason that the mechanical protection afforded by the internal brace may allow for improvement in ligamentous healing. Interestingly, IBLA use has widened with reports of use in paediatric ACL repairs (Smith et al. 2016) and Medial Collateral Ligament (MCL) repairs (Lubowitz et al. 2014).

### Dynamic Intraligamentary stabilisation (DIS)

**Fig. 2:**
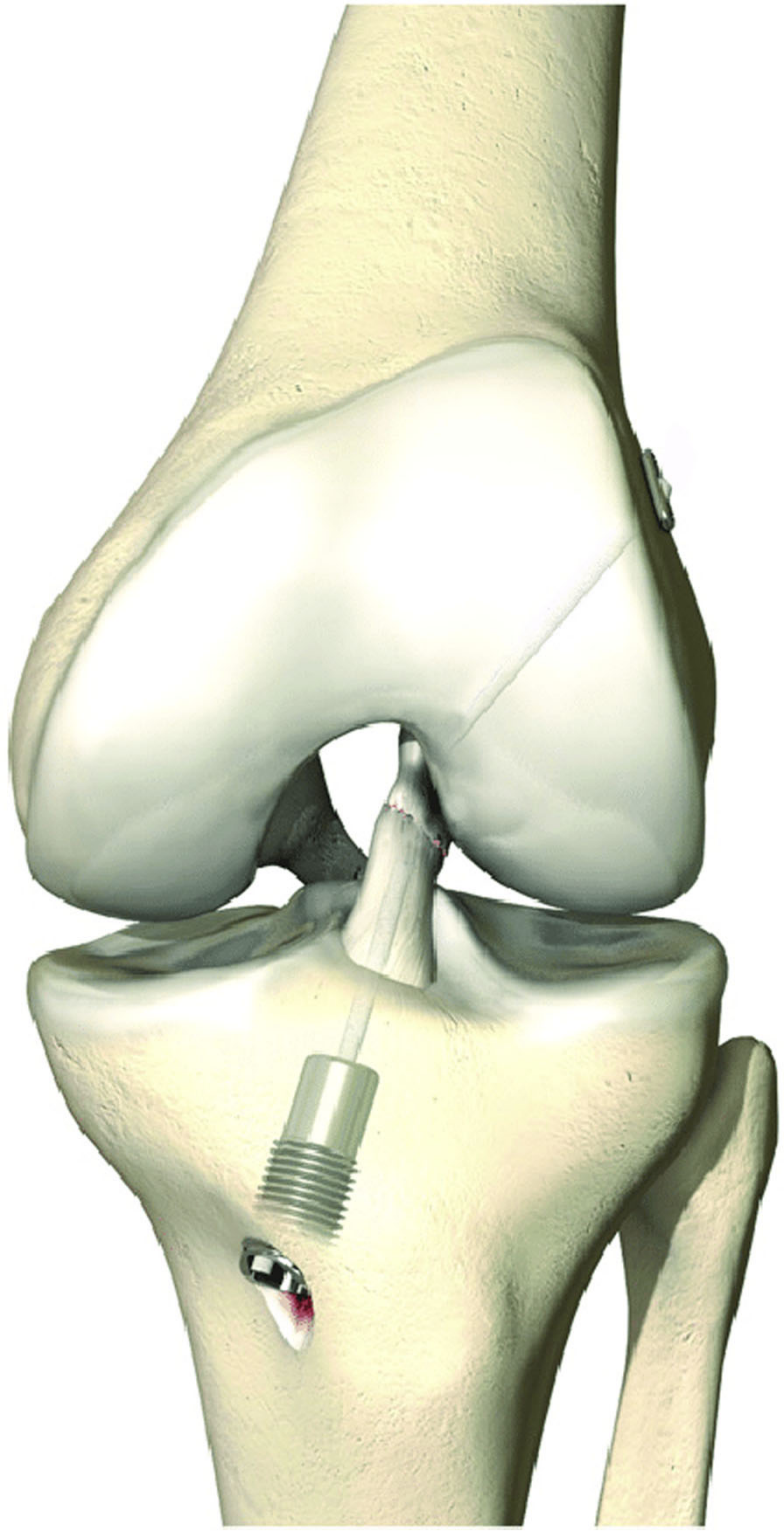
Dynamic Intraligamentary Stabilisation (Mathys Medical™)

Dynamic Intraligamentary stabilisation (DIS) (Fig. 2), developed in Berne (Switzerland), shares the concept of trying to provide a suitable protective mechanical environment in order to aid with ligamentous healing.

The procedure itself involves use of a threaded sleeve contains a preloaded spring and a mechanism for securing the spring in the tibia. A 1.8 mm braided polyethylene (PE) wire, attached to the tibial component, traverses the knee joint, through the middle of the torn ACL. It exits out of the lateral aspect of the distal femur, where it is secured with a button. Again, extensive microfracturing is performed at the femoral footprint.

The implant once inserted and tensioned applies a constant posterior drawer force to the proximal tibia. The inbuilt mechanism, by allowing 8 mm of dynamic excursion, ensures that there is a continuous tension of the cord over the entire range of motion.

Biomechanical studies in cadaveric specimens showed that DIS was capable of creating (Kohl et al. 2014) and maintaining sagittal plane stability throughout a normal rehabilitation protocol (Häberli et al. 2016). Therefore, DIS may protect the primary repair whilst allowing a full range of motion and full weight bearing immediately post-operatively in concordance with active rehabilitation protocols that have been employed so successfully in patients post ACL reconstruction.

However, in practice, initial results have been mixed thus far. Functional and objective clinical improvement with DIS has been comparable with ACL reconstruction (Henle et al. 2015; Büchler et al. 2016; Schliemann et al. 2018; Meister et al. 2018) but there is currently little evidence directly comparing DIS and ACL reconstruction with respect to improvements in donor site morbidity or proprioception.

Where DIS appears to make a difference is decreasing time from injury to surgery. Current practice is to carry out DIS in patients within 3 weeks of injury.

Not only has this led to an earlier return to work (Bieri et al. 2017) but also had the significant benefit of meniscal preservation. In a matched study (Bieri et al. 2017) meniscal intervention rates between DIS and ACL reconstruction were similar (53% vs 60% respectively) but the rate of meniscal repair (vs partial resection) was significantly higher in the DIS group (49% vs 15% respectively). The hypothesis is that early intervention prevents degradation of the meniscal tissue making it more amenable to repair. This correlates with findings of improved outcomes with early ACL reconstruction with less meniscal and chondral pathology detected in the early intervention groups (Goradia and Grana 2001; Fithian et al. 2005; Laxdal et al. 2005).

Although DIS appears to slightly expensive option there have been suggested overall cost benefits with regards to Quality Adjusted Life Years, which appear to be primarily due to the reduced costs associated with revision DIS compared with revision ACL reconstruction (Bierbaum et al. 2017).

However, multiple case series have shown early re-rupture rates appears to be in the region of 4–15%. (Henle et al. 2015, 2017; Büchler et al. 2016; Meister et al. 2018). Although it is interesting to note that these repairs are being carried out on mid-substance as well as proximal tears (Kohl et al. 2016), which we know to be less favourable to ACL repair techniques (Sherman et al. 1991; Evangelopoulos et al. 2017; Henle et al. 2017; van Eck et al. 2017; Krismer et al. 2017). The current evidence suggest that risk factors for revision surgery after DIS include younger age, higher activity level, increased AP laxity post operatively and central tear locations (Henle et al. 2017; Krismer et al. 2017), thereby making the procedure potentially unsuitable for high level athletes.

The other key issue with DIS appears to be high rates of implant removal that are required, with rates of up to 50% reported, due to local discomfort. Although interestingly these are being removed under local anaeasthetic and there may be a bias towards intervention due to the simplicity of the procedure (Häberli et al. 2018). There is no suggestion of increased failure rate with hardware removal (Bieri et al. 2017; Ateschrang et al. 2018).

Finally, there appear to be a significant requirement (1.5 to 10%) for intervention for fixed flexion deformity necessitating manipulations under anaesthetic or arthroscopic arthrolysis (1.5 to 10%) (Henle et al. 2015, 2017; Kohl et al. 2016; Bieri et al. 2017; Krismer et al. 2017; Häberli et al. 2018). It is unclear what the reasons are but extensive notch scar formation has been noted post repair and has been hypothesised as the possible cause (Ateschrang et al. 2018).

Overall, re-intervention rates appear high (40–50%) and midsubstance tears in high activity level patients pose the biggest risk for failure with DIS and currently no functional or objective clinical improvements have been noted compared with ACL reconstruction controls.

## Biologically enhanced repair

Thus far we have talked about techniques such as IBLA and DIS, which aim to aid in improving the mechanical environment. Although, initial results show an improvement from the > 50% failure rates reported by Feagin and Curl 1976, there is clearly still some room for improvement with failure rates of up to 15% seen with these new techniques. Perhaps it is more than simple mechanics that needs addressing?

We will now go on to discuss some of the techniques for so called biologically enhanced repairs, which are currently in the offing, and aim to provide biological supplementation to aid ligamentous healing.

### Bio-scaffolds

The introduction of hydrogels, over two decades ago, was one of the initial forays into the use of bioscaffolds, for use in tissue replacement or as a carrier for growth factors. They have structural similarities to the extracellular matrix of most connective tissues (Drury and Mooney 2003).

There have also been several studies looking into the use of Hyaluronan on anterior cruciate ligament healing. In an animal study, Hyaluronan was used as an intra-articular injection in partially transected ACLs in rabbits. Histologic evaluation at 12 weeks showed increased collagen type III, more angiogenesis and less inflammation in the test group and overall, an improved repair (Wiig et al. 1990). Hyaluronan has also been used to deliver growth factor for ligament healing (Berry and Green 1997) but there is no current literature to support the use of hyaluranon in vivo to supplement primary ACL repair.

Apart from Hyaluronan, engineered collagens have also been used as bio-scaffolds. Robayo et al. 2011 in an in vitro study used a tissue engineered collagen scaffold as a healing platform for ruptured ACLs. Their laboratory experiments showed colonisation of fibroblasts within the implanted collagen scaffold. In an in vivo study, on Yucatan minipigs, the use of collagen patches in ACL repairs failed to show superiority in biomechanical testing of the repaired ligaments compared to suture repair only. Histologic evaluation of the test group did not show significant differences in the Ligament Tissue Maturity Index compared to the control group (Fleming et al. 2010). However, another study from the same institute showed cruciate ligament repair augmented with collagen platelet composite patches resulted in improved biomechanical and histo-chemical characteristics of the repaired ligament (Joshi et al. 2009).

Therefore, it appears that the addition of platelets to the collagen scaffold appears to be necessary for the collagen scaffold to be effective. The collagen scaffold itself is not sufficient on its own. Interestingly, in a small case control study, addition of a collagen patch to repaired ACL midsubtance tears decreases re-rupture rate and extension deficit (Evangelopoulos et al. 2017), possible through improvements in ligament healing rates and less disordered scar formation in the notch (Ateschrang et al. 2018).

### Platelets and platelet rich plasma (PRP)

PRP has received significant attention in recent years particularly in the field of musculoskeletal pathologies. PRP has more than 3 times the normal concentration of human platelets in plasma and carries important cytokines including Platelet Derived Growth Factor (PDGF), Transforming Growth Factor (TGF) and Vascular Endothelial Growth Factor (VEGF) (Fleming et al. 2015).

Yoshida and Murray (2013), in an in vitro study, showed ACL fibroblasts that have been exposed to peripheral blood mononuclear cells and PRP for two weeks display increased cell activity in the form of proliferation, gene expression and collagen production. Cheng et al. (2010) also showed that both platelets and plasma proteins are necessary to increase collagen gene expression in fibroblasts, a necessary part of ligament healing. However, injection of PRP did not translate into improved biomechanical strength of the Anterior Cruciate Ligament repair (Murray et al. 2009).

However, Murray et al. (2006) did demonstrate that PRP combined with a collagen scaffold, resulted in improved biomechanical and histological characteristics of the repaired ACL in a canine model. In the subsequent publication, the authors demonstrated better biomechanical strength after collagen-PRP enhanced repair of porcine ACLs compared to suture repair only (Murray et al. 2007). Therefore, it appears that the collagen scaffold is an essential component in enhancing the effects of PRP. This may go beyond simply providing a mechanical scaffold as collagen causes a sustained release of anabolic cytokines such as PDGF, TGF and VEGF, which may be an additional contributory factor.

So it appears that PRP injected in to a collagen scaffold may be a suitable method of bio-enhancement. Yoshida et al. (2014) and Fleming et al. (2015) have done further work in order to identify the required platelet concentration. It appears that a platelet concentration similar to whole blood is what is required for collagen gene expression by ACL fibroblasts. Increasing platelet concentrations actually resulted in an inhibitory effect on collagen gene expression and also led to higher cell apoptosis. Therefore, platelet concentration needs to be controlled and the optimum concentration in human patients needs to be identified in order to create a solution that provides the optimum biological environment for ligamentous healing.

### Stem cells

The anterior cruciate ligament has mesenchymal stem cells (MSCs) that are mainly located close to the blood vessels and within the collagenous structure of the tissue. They have similar characteristics to bone marrow stem cells in the form of growth pattern, morphology, osteogenic and adipogenic capacity; however they are not completely identical, as MSCs originating from ACLs show less proliferation and chondrogenic capacity (Steinert et al. 2011).

Comparison of MSCs obtained from the ACL, as an intra-articular ligament, and medial collateral ligament (MCL), as an extra-articular ligament, showed significant characteristic differences. ACL stem cells showed slower growth and less differentiation potential than those from the MCL (Zhang et al. 2011). MSCs added to the natural or biodegradable scaffolds or ACL reconstruction grafts promote collagen type I and type III production within the ligament (Ge et al. 2005).

Kanaya et al. 2007 showed that intra-articular injection of cultured bone marrow MSCs in partially transected ACLs in rats accelerated healing and increased ultimate failure load of the ligament in biomechanical testing. Furthermore, Oe et al. 2011 investigated the effect of MSCs on partially transected ACLs in rats by intra-articular injection of bone marrow or mesenchymal derived stem cells. Both biomechanical and histological assessment at 4 weeks showed near normal findings and a significant improvement compared to the control group.

Numerous pre-clinical studies investigated the effects of MSCs on graft integration after ACL reconstruction. Some studies reported increased failure load and a fibro-cartilaginous zone at the bone-graft interface after ACL reconstruction in the presence of MSCs, compare to fibrous scar tissue in the control group (Ouyang et al. 2004; Lim et al. 2004; Soon et al. 2007; Matsumoto et al. 2012). In a step further, Figueroa et al. (2014) in a pre-clinical study showed 1 in 3 ACLs undergoing primary repair with collagen bio-scaffold and MSCs had complete regeneration of the ligament on histological evaluation at 12 weeks.

We have shown that there are several promising studies for bio-enhancement of ACL repairs with a combined form of PRP / MSC s/ bio-scaffold for primary intra-articular ligament repair. Murray et al. (2013) have demonstrated that this bio-enhanced repair has comparable biomechanical properties to ACL reconstruction.

Additionally and perhaps crucially, they have even shown that using a bioactive scaffold, as part of a primary ACL repair technique, can prevent post-traumatic osteoarthritis, something that has never been demonstrated with ACL reconstruction (Murray and Fleming 2013).Whether this is due to retained proprioception and therefore preserved kinematics and normal joint loading; protective growth factors released from platelets or another as yet undefined mechanism is still not known and is the focus of further work. Phase 1 trials for bridge-enhanced ACL repair have shown good results with 10 patients. Phase 2 trials are currently in progress (Bridge-Enhanced™ ACL Repair Trial).

Clearly more work needs to be done in order to translate these preliminary findings into clinical practice but it does appear that biological augmentation will have a role to play in primary ACL repair and that primary ACL repair could represent a significant advancement in the management of ACL injuries.

## Timing of surgery

If this is the case we need to consider timing of surgery. Animal studies have shown that there is a significant reduction on repair strength with delays of even 2 weeks (Magarian et al. 2010) showing a 40% decrease. This obviously poses its own obstacles when translating to clinical practice with significant delays to diagnosis and operative intervention. All of the large current case series have had ACL repair performed within 3 weeks of index injury.

Early surgery appears to facilitate meniscal preservation and earlier return to work (Bieri et al. 2017). However, there is nothing to suggest that this benefit is unique to DIS as similar benefits have been noted with early ACL reconstruction (Goradia and Grana 2001).

## Conclusions

We have used the anterior cruciate ligament as a microcosm of ligamentous repair techniques in athletic patients as it represents particular, unique challenges and difficulties when considering primary repair. Primary ACL repair has clear theoretical benefits over ACL reconstruction, particularly relevant in high performing athletes. Historical factors meant that primary repair was abandoned in favour of reconstruction and until recently has not been given much attention. However, recent work has demonstrated the potential for significant benefits with primary repair in animal models including the possible chondroprotective benefits of bio-enhanced repair techniques.

When considering suitability for primary repair it appears that femoral sided avulsions should be the initial focus, although attempts have been made to repair mid-substance tears and even a case report of a tibial sided soft-tissue avulsion being repaired (Sheth et al. 2016). Significant caution is advised with mid-substance tears as significantly higher failure rates have been shown (Sherman et al. 1991; Evangelopoulos et al. 2017; Henle et al. 2017; van Eck et al. 2017; Krismer et al. 2017).

We would also caution against using a suture repair technique alone, as they have been associated with high failure rates. We would recommend attempts be made to try and create the ideal mechanical and biological environment for healing to occur and for the repairs to be performed as acutely as possible from the time of injury. This would also appear to confer additional benefits with regards to meniscal preservation (Bieri et al. 2017).

Use of polyethylene tapes or wires that span the course of the ligament from the femur to the tibia appear to have had some initial success and it remains to be seen whether static stability with IBLA is sufficient or if a more dynamic approach, such as DIS is necessary.

It appears bio-enhancement of the ACL repair with a collagen scaffold infused with PRP or MSCs also shows some promise. There is clearly still some way to go to determine whether these techniques will translate to significant benefits for athletes, particularly as they appear to be in a higher risk group for repair failure (Henle et al. 2017; Krismer et al. 2017). However, with careful patient selection failure rates are broadly comparable with ACL reconstruction and several unanswered questions remain that provide avenues for further exploration that may yet yield benefits for repair over reconstruction e.g. how do outcomes of reconstruction post failed repair compare with outcomes post primary reconstruction?

There may well be a new frontier on the horizon for the treatment of ACL injuries but it may be the non-athlete that leads the way.

## Abbreviations

ACL: Anterior cruciate ligament
DIS: Dynamic Intraligamentary Stabilisation
IBLA: Internal Brace Ligament Augmentation
MCL: Medial collateral ligament
MRI: Magnetic resonance imaging
MSC: Mesenchymal stem cell
PDFG: Platelet derived growth factor
PE: Polyethylene
PRP: Platelet rich plasma
TGF: Transforming growth factor
VEGF: Vascular Endothelial Growth Factor
